# Metabolism of Caprine Milk Carbohydrates by Probiotic Bacteria and Caco-2:HT29–MTX Epithelial Co-Cultures and Their Impact on Intestinal Barrier Integrity

**DOI:** 10.3390/nu10070949

**Published:** 2018-07-23

**Authors:** Alicia M. Barnett, Nicole C. Roy, Adrian L. Cookson, Warren C. McNabb

**Affiliations:** 1 Food Nutrition & Health Team, AgResearch Ltd., Palmerston North 4442, New Zealand; nicole.roy@agresearch.co.nz; 2 Riddet Institute, Massey University, Palmerston North 4442, New Zealand; adrian.cookson@agresearch.co.nz (A.L.C.); W.Mcnabb@massey.ac.nz (W.C.M.); 3 High-Value Nutrition National Science Challenge, Auckland 1142, New Zealand; 4 Food Assurance Team, AgResearch Ltd., Hopkirk Institute, Palmerston North 4474, New Zealand

**Keywords:** caprine milk carbohydrates, in vitro studies, small intestinal epithelium, barrier integrity, probiotic lactobacilli bacteria

## Abstract

The development and maturation of the neonatal intestine is generally influenced by diet and commensal bacteria, the composition of which, in turn, can be influenced by the diet. Colonisation of the neonatal intestine by probiotic *Lactobacillus* strains can strengthen, preserve, and improve barrier integrity, and adherence of probiotics to the intestinal epithelium can be influenced by the available carbon sources. The goal of the present study was to examine the role of probiotic lactobacilli strains alone or together with a carbohydrate fraction (CF) from caprine milk on barrier integrity of a co-culture model of the small intestinal epithelium. Barrier integrity (as measured by trans epithelial electrical resistance (TEER)), was enhanced by three bacteria/CF combinations (*Lactobacillus rhamnosus* HN001, *L. plantarum* 299v, and *L. casei* Shirota) to a greater extent than CF or bacteria alone. Levels of occludin mRNA were increased for all treatments compared to untreated co-cultures, and *L. plantarum* 299v in combination with CF had increased mRNA levels of *MUC4*, *MUC2* and *MUC5AC* mucins and MUC4 protein abundance. These results indicate that three out of the four probiotic bacteria tested, in combination with CF, were able to elicit a greater increase in barrier integrity of a co-culture model of the small intestinal epithelium compared to that for either component alone. This study provides additional insight into the individual or combined roles of microbe–diet interactions in the small intestine and their beneficial contribution to the intestinal barrier.

## 1. Introduction

Milk is the first food for all mammals [1] and provides the nutritional needs for normal growth and development of the rapidly growing offspring [2]. Milk has a complex chemical composition which can be influenced by the diet, environmental conditions and the stage of lactation, and can also vary between animal species [1]. For example, the average protein, lipid and lactose profile of human milk is 1.2%, 4.4% and 6.8%, respectively, whilst that of caprine milk is 3.4%, 3.9% and 4.4%, respectively [2]. Together with lactose, oligosaccharides are the main contributor to the carbohydrate profile of milk, and in human milk there are 5–8 g/L of oligosaccharides [3]. In comparison caprine milk has 0.25–0.3 g/L of oligosaccharides, which although less than human milk, has an overall profile of neutral and acidic oligosaccharide structures which is more similar to human milk than either bovine or ovine milk [3].

Milk in early age has multifunctional roles beyond simply nutrition within the gastrointestinal tract by assisting in the establishment of a symbiotic microbiota [1], aiding in the development of the immune system, stimulating cellular growth and inducing epithelial barrier maturation [4]. At birth, although the gastrointestinal tract has all the cellular and structural features of the adult intestine [5], the epithelial barrier is functionally immature, and as such, there is increased risk of injury and inflammation caused by the movement of toxins and bacteria from the lumen [6]. The intestinal barrier is comprised of a single layer of epithelial cells known as the intrinsic barrier and the extrinsic mucus barrier [7,8,9]. Important components of the intrinsic barrier are the intercellular junctional complexes [10]. Occludin is an integral membrane protein that contributes to the tight junction (TJ) complex and interacts with tight junction protein (TJP)-1 and TJP2, which in turn interacts with the cytoskeleton [11]. The TJ structure can be altered in response to stimuli, such as growth factors, pathogenic/commensal bacteria, and dietary components leading to an increase or decrease in permeability [12]. Dietary components, such as fructo-oligosaccharides, can directly promote barrier protective effects by activating host cell signalling and the induction of select TJs in the intestine [13].

Intestinal development and barrier maturation after birth is not only influenced by dietary intake, but also by the colonisation by mutualistic microorganisms [14]. Bacterial colonisation is initiated in utero [15], but establishment of the intestinal microbiota after birth is influenced by the mode of delivery and the environment [16]. The establishment of a microbiota that is protective to the infant [17] may aid in barrier integrity via modulation of host gene expression and mucin secretion [18,19,20], whereas abnormal bacterial colonisation may disrupt this process and contribute to the development of host diseases [21]. Diet plays a major role in selecting for initial colonisers of the intestine [17], for example; milk oligosaccharides which are composed of 3–10 monosaccharide residues [22] are not-digested by infants, but instead pass unabsorbed into the large intestine where they selectively stimulate the growth and/or activity of specific bacterial genera such as bifidobacteria and lactobacilli [23,24]. In addition to the beneficial effects exerted by such oligosaccharides on the commensal microbiota, they are generally considered to improve the survival, adherence, transient colonisation and subsequent proliferation of probiotic micro-organisms [25,26]. However, few reports have focused on the influence of such substrates on the adherence of probiotic bacteria in the small intestine.

Determining the adherence or persistence of dietary probiotics in the intestine is largely dependent on measurements of the microbial composition in faecal samples, mainly because obtaining samples directly from the small intestine is difficult due to the highly invasive intubation methods used. At best the measurement in faecal samples reflects the microbial composition of the large intestine, which differs substantially from that of the small intestine. For example, anaerobic bacteria from the families Bacteriodaceae and Clostridiaceae are found in high abundance in the large intestine [27], whilst in the small intestine, fast-growing facultative anaerobes such as Lactobacillaceae and Enterobacteriaceae are dominant families of the microbial community, which “tolerate the combined effects of bile acids and antimicrobials while still effectively competing with both the host and other bacteria for the simple carbohydrates that are available in this region of the gastrointestinal tract” [27]. Studies of orally administered lactic acid bacteria have demonstrated that the lactic acid bacterial counts in the small intestine increase after ingestion [28,29,30,31]. Evidence indicates that particular *Lactobacillus* probiotic strains can strengthen and preserve the intestinal barrier in an in vitro model of necrotising enterocolitis [32], increase the expression of genes involved in TJ formation [33], and increase barrier integrity of intestinal epithelial cell (IEC) monolayers as measured by trans epithelial electrical resistance (TEER) [34,35,36]. In addition, probiotics may provide protective effects for the intestinal barrier by actively secreting soluble mediators [37], facilitating TJ formation [38], and inducing mucin gene expression with a resultant change in the mucus layer composition which may occur as a direct response to bacterial adhesion to the epithelium [39]. From in vitro investigations using IECs such as mono-cultures of Caco-2 cells [34], mono-cultures of HT29 (and various sub-clones) [40], and co-cultures of Caco-2:HT29–MTX cells [41] it has been determined that adherence of probiotics can be both species and strain specific [42] and dependent on the carbon source present in their growth medium [43]. For example, a study undertaken by Wickramasinghe et al. [44] determined that there was a higher rate of adhesion of *Bifidobacterium infantis* ATCC 15697 to Caco-2 cells when grown with human milk oligosaccharides than when cultured in lactose [44]. These studies provided the evidence of the protective effects of some probiotics on epithelial barrier maturation and the ability of prebiotic substrates to enhance the survival and transient colonisation of probiotic bacteria in the intestinal tract.

In this study, we hypothesised that a carbohydrate fraction (CF) from caprine milk in combination with known probiotic bacterial strains would increase the barrier integrity of a co-culture model of the small intestinal epithelium when compared to either the CF or bacteria alone. A representative co-culture model of the small intestine which incorporated both absorptive enterocytes (Caco-2) and mucin secreting goblet cells (HT29–MTX), in a ratio similar to that of the small intestine (90:10 Caco-2:HT29–MTX) [45] was used because this would typically be the first site of probiotic interaction with the host’s intestinal cells and the site at which the host and the microbiota (probiotics) compete for the simple carbohydrates found in milk such as lactose, glucose and galactose. Additionally, because milk oligosaccharides are not absorbed or directly utilised by cells of the small intestine, these components of milk have the potential to influence the adherence and therefore the overall persistence of probiotic bacterial strains in the small intestine. The epithelial barrier integrity (as measured by TEER), the expression levels of genes that encode for TJs and mucins, and the abundance of mucin proteins were measured after 3 h, to determine if selected probiotic bacterial strains in combination with CF had a greater enhancing effect on barrier integrity, when compared to either component alone. A 3 h time point was used in this study as this reflects the transit time of digesta through the small intestine (15 min to 5 h) and as such is biologically relevant. A comparison of the effects on the barrier integrity of the co-culture model could help elucidate the distinct responses of intestinal cells to the combined or specific effects of probiotics or the CF.

## 2. Materials and Methods 

### 2.1. Composition of Carbohydrate Fraction (CF) and Stock Solutions

The CF used in this study was kindly provided by Caroline Thum (AgResearch, Grasslands, Palmerston North, NZ) [46]. The carbohydrate composition of the CF (as a percentage of total carbohydrates) used in this study was: 25.6% oligosaccharides, 0.4% galacto-oligosaccharides, 46.1% lactose, 12% glucose and 15.9% galactose [47].

In addition to CF, a sugar combination (galactose, glucose and lactose (all from BDH, Global Science, Auckland, NZ)) as well as the monosaccharide galactose and the disaccharide lactose were used at comparable concentrations to that found in the CF. Also two acidic oligosaccharides, 3′ and 6′ sialyl lactose (both from Carbosynth, Berkshire, UK) were used.

The CF and selected carbohydrates for all experiments were suspended in phosphate buffered saline (PBS, pH 7.2), and filter sterilised (0.22 µm filters; Millipore Australia Pty Ltd., Sydney, Australia). For use in IEC assays, stock carbohydrate solutions were diluted with Dulbecco’s Modified Eagles Medium (DMEM; Life Technologies, Penrose, Auckland, NZ). CF was used at a final concentration of 4 mg/mL because this concentration of CF has previously been shown to increase TEER, and *MUC2* and *MUC5AC* mucin gene and protein abundance of 90:10 Caco-2:HT29–MTX co-cultures [47]. The sugar combination, galactose and lactose were used at final concentrations (comparable to those found in the CF) of 3 mg/mL, 0.6 mg/mL and 1.8 mg/mL respectively, whilst 3′ and 6′ sialyl lactose were both used at 1 mg/mL.

### 2.2. IEC Co-Culture Conditions

The human colon adenocarcinoma cell line HT29 (HTB-38; ATCC, Manassas, VA, USA) previously adapted with 10^−7^ M methotrexate (MTX) was kindly provided by Rachel Anderson (AgResearch, Grasslands, Palmerston North, NZ) and further adapted with 10^−6^ M MTX as described previously [48,49]. The human colorectal adenocarcinoma cell line Caco-2 (HTB-37) was obtained from the ATCC at passage 18. The HT29–MTX and Caco-2 cells were used in experiments from passage 18–25 and 28–33 respectively.

Caco-2 and HT29–MTX cells were cultured separately in tissue culture flasks (Corning, Lindfield, Sydney, Australia) in DMEM supplemented with 10% (*v*/*v*) foetal bovine serum (FBS; Life Technologies, Auckland, NZ) and 1% (*v*/*v*) Penicillin-Streptomycin (Pen-Strep; 10,000 units/mL Penicillin and 10 mg/mL Streptomycin; Sigma-Aldrich, Auckland, NZ) as described previously [47].

Both Caco-2 and HT29–MTX cells were subcultured when they reached 80% confluence, and re-seeded at a 1:5 dilution into new 75 cm^2^ flasks (Corning). The cultures were maintained at 37 °C in a 5% CO_2_, 95% air/water saturated atmosphere, with the medium being replaced every 48 h.

For experimental studies, Caco-2 and HT29–MTX cells were stained with trypan blue, counted using the Countess automated cell counter (Life Technologies), suspended at a ratio of 90:10 (Caco-2:HT29–MTX) to simulate the cellular configuration of the small intestine [45] and seeded at a density of 6.3 × 10^4^ cells per cm^2^. Co-cultures of IECs were cultured for 21 days. Twenty four hours prior to their use in experiments (day 20 post-seeding) the co-cultures were washed as described previously [47], replenished with serum- and antibiotic-free medium (DMEM) to eliminate any interference from extraneous proteins or hormones [50] and incubated for 24 h.

### 2.3. Bacterial Strains and Culture Conditions

Four probiotic lactobacilli strains used in this study were originally isolated from human, dairy or food origins; *L. rhamnosus* Goldin and Garbach (LGG; American Type Culture Collection (ATCC) 53103—healthy human faecal sample)*, L. plantarum* 299v (Lp299v; Deutsche Sammlung von Mikroorganismen (DSM) 9843—healthy human intestinal mucosa), *L. rhamnosus* HN001 (HN001; Danisco New Zealand Ltd, Auckland, NZ—dairy—cheddar cheese), and *L. casei* Shirota (LcS; Yakult New Zealand, Auckland, NZ—food). These strains were chosen as they are all Generally Recognised As Safe (GRAS) by the United States Food and Drug Administration (FDA) and as such have published data showing their efficacy in in vitro models, animal models, and controlled human trials. For more information refer to the FDA website using the GRAS notice numbers: 231 (LGG), 685 (Lp299v), 288 (HN001), and 429 (LcS). Strains were stored in deMan, Rogosa and Sharpe (MRS) broth (Acumedia, MI, USA) containing 35% glycerol at −80 °C and propagated twice in MRS broth prior to use. All strains were grown overnight at 37 °C in anaerobic broth (MRS flushed with oxygen-free CO_2_) using Hungate culture tubes (16 mm diameter, 125 mm long; BellCo glass, Vineland, NJ, USA) sealed with butyl rubber stoppers. For all studies, the bacterial strains were used at stationary growth phase. The time taken for bacteria to enter stationary phase was determined by constructing growth curves by measuring optical density at 600 nm (OD600) using an Ultrspec 1100 pro photometer (Amersham Biosciences, Auckland, NZ) at intervals during growth.

### 2.4. Bacterial Growth with CF and Selected Carbohydrates

The growth characteristics of each probiotic were assessed in triplicate using Hungate tubes containing DMEM-supplemented with the selected carbohydrate substrate. DMEM was used as the basal media because it is the same media that the Caco-2:HT29–MTX (90:10) co-cultures are typically cultured with. Briefly, 30 µL of stationary phase bacteria from MRS broth cultures was inoculated into the test media. All cultures were incubated in a 5% CO_2_, 95% air/water saturated atmosphere at 37 °C. The growth of the bacteria was monitored by measuring optical density at 600 nm at hourly intervals post inoculation, and assessed after 3 h. Prior to absorbance measurements the incubated tubes were inverted three times to suspend any sedimented bacterial cells. The arithmetical median was calculated from all single OD readings and absorbance values at time zero were subtracted from each time point for respective Hungate tubes.

### 2.5. IEC and Bacterial Cell Co-Cultures

Bacteria from stationary phase cultures were washed by diluting 1:5 in PBS, following centrifugation at 2492× *g* for 5 min (11180/13190 rotor, Sigma 3-18K centrifuge) and re-suspension in DMEM or DMEM supplemented with CF. Approximately 10^7^ colony forming units (CFU) were added to each well as ascertained by plate counts. 

Caco-2:HT29–MTX (90:10) co-cultures in 24-well tissue-culture plates (Corning), were prepared 24 h prior (day 20 post-seeding) to the adhesion assay as described in Section 2.2. On the day of the assay the co-cultures were gently washed four times with PBS, and to appropriate wells, bacteria-supplemented medium was added and plates incubated at 37 °C in a 5% CO_2_ atmosphere for 3 h.

### 2.6. Adhesion Assays to IEC Co-Cultures

Following incubation, the growth medium was removed, and the co-cultures were gently washed 4 times with PBS to remove any non-adherent bacteria, and lysed with 1 mL of PBS containing 1% (*v*/*v*) Triton X-100 (Sigma-Aldrich) to release adherent bacteria. The lysates were serially diluted with PBS and bacteria enumerated on MRS agar plates as described previously [34]. To determine original CFU/mL aliquots of the experimental inocula were retained, diluted, and plated on MRS agar. To account for variaions in the orginal inocula between strains the results were expressed as adherent bacteria as a percentage of the original inoculum, (CFU/mL of recovered adherent bacteria ÷ CFU/mL of inoculum) × 100. Each adhesion assay was conducted independently in triplicate over two successive passages of IECs.

### 2.7. Measurement of Metablic Activity of IEC Co-Cultures

The metabolic activity of Caco-2:HT29–MTX (90:10) co-cultures was quantified by absorbance at 450 nm with a reference wavelength of 650 nm (FlexStation 3 Benchtop Multi-Mode Microplate Reader; Molecular Devices, Sunnyvale, CA, USA) using the 4–[3–(4–Iodophenyl)–2–(4–nitrophenyl)–2H–5–tetrazolio]–1,3 benzene disulfonate (Wst-1) colourimetric assay (Roche, Auckland, NZ). In 96-well tissue-culture plates (Corning), post-confluent (21 days post-seeding) Caco-2:HT29–MTX (90:10) co-cultures were incubated for 3 h in carbohydrate-supplemented DMEM media. Additional wells in each plate containing medium and Wst-1 reagent only (without cells) were processed in parallel and used as reference blanks [51,52]. Each substrate was tested in 10 replicates, over three successive passages of IECs. The metabolic activity of the Caco-2:HT29–MTX (90:10) co-cultures was expressed as absorbance (A450 nm–A650 nm).

### 2.8. TEER Assay

The TEER assays were undertaken as described in [47]. Briefly, Caco-2 and HT29–MTX cells (90:10) were seeded onto 12 mm diameter, 0.4 µm^2^ pore size, polyester (PET) Transwell inserts (Corning) and cultured as described in Section 2.2.

Post-confluent, differentiated co-cultures were prepared 24 h prior (day 20 post-seeding) to the TEER assay as described in Section 2.2. After 24 h incubation, initial resistance readings were obtained (EndOhm Culture cup connected to an EVOM voltohmmeter (World Precision Instruments, Sarasota, FL, USA)) for all co-cultures. The medium in the well was replaced with DMEM, and the medium in the Transwell insert was replaced with either DMEM (untreated) or DMEM supplemented with CF (4.0 mg/mL) with or without bacteria. The resistance across each cell monolayer was measured after 3 h, and the percentage change in TEER calculated as described previously [34]. Experiments were undertaken in triplicate (three successive passages of cells), each with three replicates per treatment.

### 2.9. Mucin Protein Quantification

The abundance of mucin proteins in cell lysate (CL) and spent media (SM) was determined by indirect enzyme linked immunosorbent assay (indirect ELISA) using MUC2 mouse mono-clonal antibody (clone 4A4, 1:250; Creative Biomart, New York, NY, USA), MUC4 mouse mono-clonal antibody (clone 5B12, 1:500; Abnova, Taipei, Taiwan) or MUC5AC mouse mono-clonal antibody (clone 2H7, 1:250; Abnova) and horseradish peroxidase-rabbit anti-mouse immunoglobulin G conjugate (Abcam, Cambridge, UK; 1:5000 dilution for MUC2 and MUC5AC and 1:10,000 dilution for MUC4) as described previously [47]. 3,31,5,51-tetramethylbenzidine (TMB) peroxidase solution (Invitrogen) was added for 0.5 h and stopped with 2N H_2_SO_4_ (Reagent grade sulphuric acid; Sigma-Aldrich). Using the well scan option on a FlexStation 3 Benchtop Multi-Mode Microplate Reader (Molecular Devices, Sunnyvale, CA, USA) the absorbance was read at 450 nm and the abundance of mucin proteins was calculated from standard curves using MUC2 (Creative Biomart, New York, NY, USA), MUC4 (Abnova) and MUC5AC (Abnova) recombinant proteins as standards. Experiments were undertaken in triplicate (three successive passages of cells), each with three replicates per treatment. Each sample was analysed in duplicate by indirect ELISA.

### 2.10. Quantification of mRNA of IEC Co-Cultures

The expression of mucin and TJ related genes in Caco-2:HT29–MTX (90:10) co-cultures was quantified using TaqMan quantitative real-time PCR (qPCR). All reagents were obtained from Applied Biosystems (Foster City, CA, USA) unless otherwise stated. The expression of these genes in reference samples (untreated controls) was also quantified. The genes quantified were; *MUC2*, *MUC4*, *MUC5AC*, *TJP1*, *TJP2*, and *OCLN*. (TaqMan assay IDs Hs.PT.56a.26485553, Hs.PT.56a.5039491, Hs.PT.56a.25473826, Hs.PT.58.39733148, Hs.PT.58.25666947 and Hs.PT.58.24465876 respectively).

Caco-2:HT29–MTX cells (90:10) were seeded into 12-well cell culture plates (Corning). Twenty days post-seeding monolayers were prepared as described in Section 2.2 After 24 h the SM was removed and monolayers washed gently four times with PBS. Pre-warmed, DMEM (untreated) or DMEM supplemented with 4.0 mg/mL CF was gently added to the monolayers either with or without the bacterial strains of interest and cultures incubated for 3 h.

After 3 h the SM was removed and monolayers lysed with 1 mL of Tri-reagent (Invitrogen). The total RNA from each well was isolated as described previously [47] using the RiboPure RNA isolation kit. The total RNA was stored at −80 °C overnight and quantified using a Nanodrop 1000 spectrophotometer (Thermo Fisher Scientific, Auckland, NZ). The integrity of the RNA was measured using an Agilent 2100 Bioanalyser (Agilent Technologies, Santa Clara, CA, USA) to ensure samples had an RNA integrity number (RIN) above 8.0 prior to downstream analysis.

For real-time PCR analysis, 1.5 µg of total RNA was reverse transcribed into cDNA using a high-capacity RNA-to-cDNA Kit (Applied Biosystems) according to the manufacturer’s instructions. The cDNA was stored at −20 °C prior to the determination of the expression levels of the six genes, relative to the reference genes hypoxanthine phosphoribosyltransferase [53,54] (*HPRT1*; Hs.PT.39a.22214821), glyceraldehyde 3-phosphate dehydrogenase (*GAPDH*; Hs.PT.39a 22214836) and βeta-2-microglobulin (*B2M*; Hs.PT.58v.18759587) determined using TaqMan probes on the Rotor-Gene 6000 real-time thermal cycler (Corbett Life Science, Concord, Australia). The *ACTB* (Hs.PT.39a.22214847) reference gene was also evaluated but excluded from the final analysis as it did not meet the requirements of a reference gene in all the samples tested [55]. All PCRs (no template controls, untreated, and treated samples) were prepared as triplicate 10 µL reactions as described previously [55]. The thermal profile used was 95 °C for 180 s followed by 40 cycles 95 °C for 3 s and 60 °C for 30 s. The data were normalised to the reference genes and analysed for expression level changes using Relative Expression Software Tool (REST) 2009 software (version 2.0.13; Qiagen, Valencia, CA, USA). Experiments in triplicate were completed (three successive passages of cells), each with three replicates per treatment. Each sample was analysed in triplicate by qPCR.

### 2.11. Statistical Analysis

Data were first evaluated for normality with the Shapiro-Wilk test, and for equal variance with the Brown-Forsythe test using SigmaPlot 13.0b software. Data that were normally distributed but had heterogeneous variances, such as the bacterial growth and adherence data, were assessed by non-parametric tests, namely the Kruskall–Wallis test, followed by the Mann–Whitney U test. All TEER assay data were analysed for statistical significance, using a repeated measure ANOVA with SigmaPlot 13.0b software. The real-time PCR data was analysed using REST with efficiency correction [45]. For the Wst-1 metabolic activity bioassay and the ELISA protein abundance assay, treatments were compared using an analysis of variance (ANOVA), followed by the Holm–Sidak post-hoc method. Differences were considered statistically different at probability values less than 0.05.

## 3. Results and Discussion

The aim of this study was to determine the influence of specific combinations of probiotic bacteria and a CF from caprine milk on the barrier integrity of IEC co-cultures. The CF used in this study was a mixture of carbohydrates therefore we first sought to determine whether CF or individual CF components had contrasting impacts on the growth of four probiotic bacteria or the metabolic activity of the IECs. A pure preparation of oligosaccharides could not be obtained using our standard CF purification methods, therefore we used the acidic milk oligosaccharides 3′ and 6′ sialyl lactose as they represent 22% of the oligosaccharides found in the CF [47].

### 3.1. Selective Carbohydrate Fermentation by Probiotic Lactobacilli

**Figure 1:**
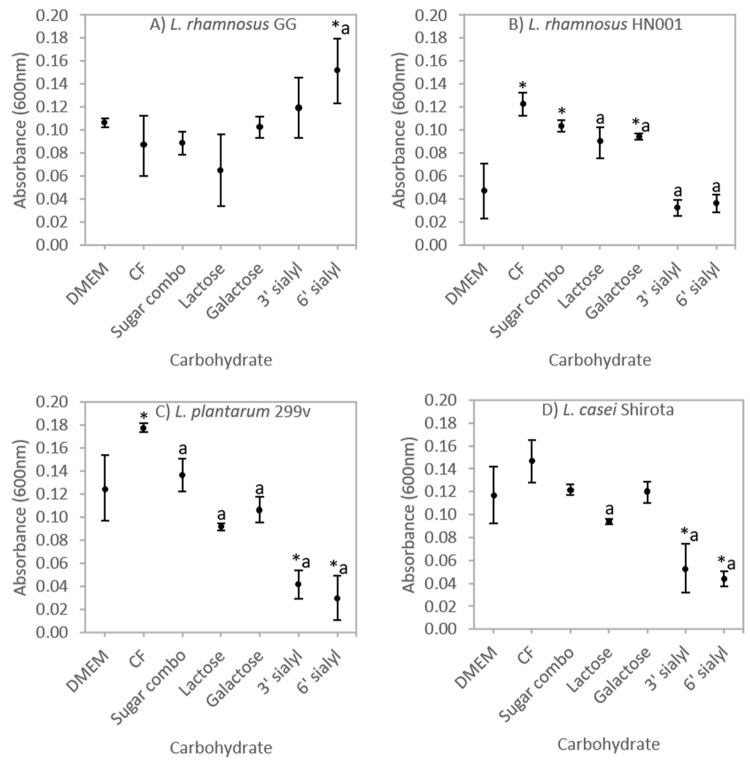
Ability of (**A**) *Lactobacillus rhamnosus* GG, (**B**) *L. rhamnosus* HN001, (**C**) *L. Plantarum* 299v, and (**D**) *L. casei* Shirota to ferment carbohydrates. Bacterial growth (absorbance 600 nm) in Dulbecco’s Modified Eagles Medium (DMEM) or DMEM supplemented with a carbohydrate fraction from caprine milk (CF; 4 mg/mL) or selected carbohydrates (at comparable concentrations to those found in the CF—refer to text) as fermentable carbohydrate source as indicated and cultured for 3 h under 5% CO_2_ atmospheric conditions. Values represent the mean absorbance (±S.D.); *n* = 3. * = significantly different (*P* < 0.05) to DMEM media control; *a* = significantly different (*P* < 0.05) to CF.

The effect of carbohydrate substrate on the growth of all four probiotic *Lactobacillus* strains when cultured in DMEM (5% CO_2_) was examined (Figure 1A–D). Only *L. rhamnosus* GG appeared able to utilise the acidic milk oligosaccharide 6′ sialyl lactose for growth compared to both the media control (DMEM) and CF (Figure 1A). The growth of the remaining three lactobacilli strains during culture in 3’ and 6′ sialyl lactose supplemented media was significantly lower than CF (Figure 1B–D).There was increased growth of *L. rhamnosus* HN001 during incubation with CF, sugar combination and galactose compared to the DMEM control (Figure 1B). Similarly, growth of *L. plantarum* 299v in CF was increased when compared to all other carbohydrate substrates investigated (Figure 1C) suggesting that *L. plantarum* 299v could utilise additional components such as the neutral oligosaccharide *N*-Acetyl-glucosaminyl-lactose or other acidic oligosaccharides that are present in the CF but not in the sugar combination. *L. plantarum* strain FUA3112 has previously been reported to metabolise neutral oligosaccharides [56].

### 3.2. Carbohydrates Do not Influence Metabolic Activity of IEC Co-Cultures

**Figure 2:**
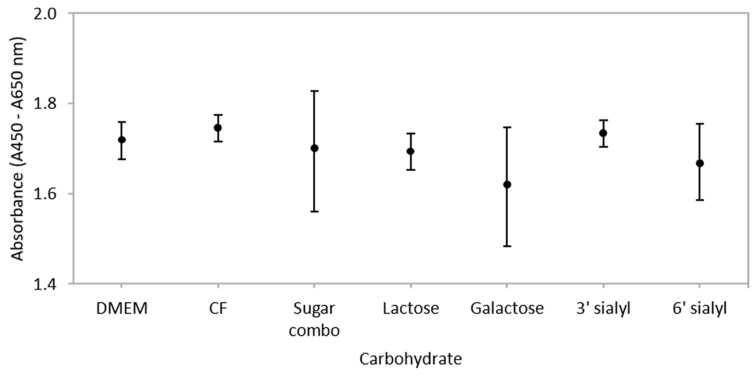
The metabolic activity of post-confluent (21 days post seeding) 90:10 Caco-2:HT29–MTX co-cultures after 3 h incubation with a carbohydrate fraction (CF) from caprine milk (CF; 4 mg/mL) and selected carbohydrates (at comparable concentrations to those found in the CF—refer to text) as determined from the Wst-1 assay. Values are means (±SEM) for three experiments (10 samples per treatment per experiment); *n* = 3. DMEM is the media control.

Incubation of monolayers with CF or selected carbohydrates for 3 h had no significant effect on the metabolic activity and thus proliferation rates, of post-confluent Caco-2:HT29–MTX (90:10) co-cultures when compared to the media control (DMEM) (Figure 2). A previous study by Kuntz et al. [57] reported that proliferation rates of HT29 and Caco-2 cells was inhibited by neutral oligosaccharides (2.3 and 0.2 mg/mL respectively) and acidic oligosaccharide (0.3 and 0.7 mg/mL respectively) isolated from human milk [57]. In contrast, 1 mg/mL lactose had no effect on the proliferation of primary human foetal intestinal cells [58], but proliferation of the same cell line was increased after exposure to whey produced from bovine colostrum [59] and complete human milk [60]. Thus, it could be suggested that treatment of IECs with purified preparations of neutral or acidic oligosaccharides inhibits cellular proliferation to a greater extent than IECs exposed to treatments comprised of a combination of carbohydrates such as the CF. How this relates to the mechanism of action was not determined in this study, but it is known that neutral and acidic oligosaccharides inhibit proliferation rates of IECs through alterations in epidermal growth factor receptor (EGFR) signalling and cell cycle regulators [58,61], and human milk increases proliferation through a unique tyrosine kinase pathway [60].

### 3.3. CF Modulates Bacterial Adherence to Caco-2:HT29–MTX (90:10) Co-Cultures

**Figure 3:**
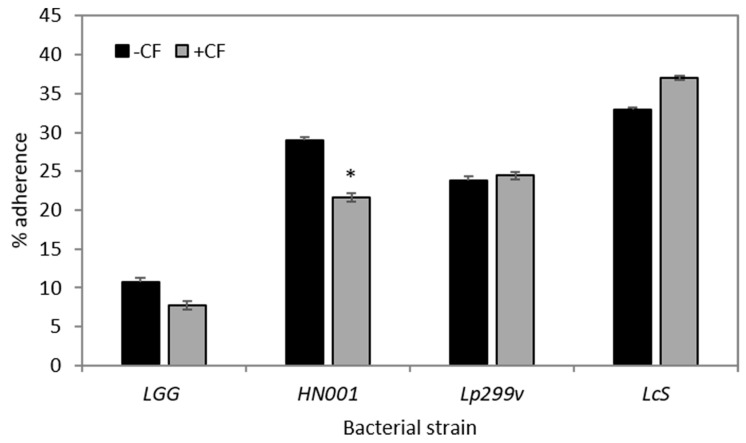
Influence of a carbohydrate fraction (CF) from caprine milk (CF; 4 mg/mL) on the adherence of bacteria (as percentage of inoculum) to 90:10 Caco-2:HT29–MTX co-cultures. Data are expressed as the means (±SEM) for three experiments (three samples per treatment per experiment); *n* = 3. * = Significantly different (*P* < 0.05) to respective bacteria only treated monolayers. LGG = *L. rhamnosus* GG; HN001 = *L. rhamnosus* HN001; Lp299v = *L. plantarum* 299v and LcS = *L. casei* Shirota.

There was a marked species-specific difference in adherence of the four probiotic bacterial strains to Caco-2:HT29–MTX (90:10) co-cultures (Figure 3). This result is similar to that observed previously [62]. Although levels of adherence were modulated by the inclusion of CF in the media, only the adherence of *L. rhamnosus* HN001 was significantly reduced (Figure 3). Decreased adherence to the epithelial monolayers in CF-supplemented assay media, such as occurred for *L. rhamnosus* HN001, may have been related to the oligosaccharides in the CF being structurally similar to receptor sites of the IECs or mucus layer to which specific bacteria recognise and adhere. For some bacterial strains, oligosaccharides may act as a molecular receptor decoy inhibiting bacterial adherence [63]. Unfortunately the binding of oligosaccharides to the surface of probiotic bacteria or commensal bacteria is not well investigated, although there is a larger body of work studying glycan binding with pathogenic bacteria [64].

Other strain specific observations have been reported previously for *Lactobacillus* strains; Kadlec et al. [25] reported that the prebiotic substance Orafti P95 increased the adherence of *L. rhamnosus* strain CCDM150 to Caco-2:HT29–MTX (90:10) co-cultures, but decreased the adherence of both *L. rhamnosus* strain CCDM289 and strain CCDM598 to the same co-cultures [25].

Adherence to intestinal surfaces, whether directly to IECs or the mucus layer, and temporary colonisation within the intestine, are considered to be defining activities of a probiotic [62]. For *Lactobacillus* species, both protein and non-protein mediated adherence mechanisms have been reported [65]. For example, *L. rhamnosus* GG possesses mucin binding proteins [66] which are known as one of the effector molecules involved in its adherence to the host [67]. Conversely, the cell surface of *L. rhamnosus* GG contains high molecular weight, galactose rich heteropolymeric exopolysaccharide molecules, which may negatively impact its adherence, possibly by shielding adhesion molecules [68,69]. The binding affinity of bacteria can be modulated by the presence of different sugars on the epithelial surface. For example *L. plantarum* 299v adheres to mannose residues on IECs [70,71]. Adherence of bacteria to biotic or abiotic surfaces can also be influenced by growth temperature, pH of the cultures and the specific growth phase of the bacteria themselves [72,73].

### 3.4. The Combination of CF and Probiotic Bacteria Increases TEER

**Figure 4:**
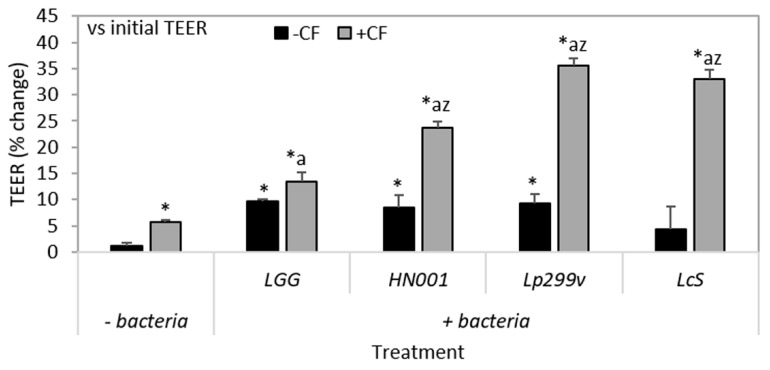
Influence of a carbohydrate fraction (CF) from caprine milk (CF; 4 mg/mL) and probiotic bacteria either alone or in combination on trans-epithelial electrical resistance (TEER) of 90:10 Caco-2:HT29–MTX co-cultures. The change in TEER as the percentage change after 3 h compared with initial TEER. Values are means (±SEM) for three experiments (three samples per treatment per experiment); *n* = 3. * = Significantly different (*P* < 0.05) to untreated monolayers; *a* = significantly different (*P* < 0.05) to CF treated monolayers; and z = significantly different (*P* < 0.05) to respective bacteria only treated monolayers. UNT = untreated; LGG = *L. rhamnosus* GG; HN001 = *L. rhamnosus* HN001; Lp299v = *L. plantarum* 299v and LcS = *L. casei* Shirota.

After 3 h incubation the TEER of Caco-2:HT29–MTX (90:10) co-cultures was increased for all individual bacteria/CF combinations compared to untreated, CF and also to the respective bacteria only control, except for the *L. rhamnosus* GG/CF combination (Figure 4). Only co-cultures treated with *L. casei* Shirota had similar TEER compared to untreated (Figure 4).

Similar to that of bacterial adherence, the effect of bacteria/CF combinations on TEER was strain-dependent (Figure 4). This observation is concordant with other studies where *L. plantarum* strain 299 had a greater effect on enhancing the TEER of Caco-2 cells compared to *L. plantarum* strain 299v [34]. In addition, a previous study using Caco-2 mono-cultures, determined that there were definite species- and sugar-dependent effects with fermentation of inulin based oligofructose [74,75] where the probiotic *B. lactis* Bb-12 exerted the most beneficial effects [75]. In another study, the synbiotic combination of resistant starch and *B. lactis* (strain unknown), protected against the development of colorectal cancer in rats, and was greater than the benefit of either component alone [76].

### 3.5. CF and Bacteria When Cultured Alone or in Combination Impacts on TJ Related Gene Expression

**Figure 5:**
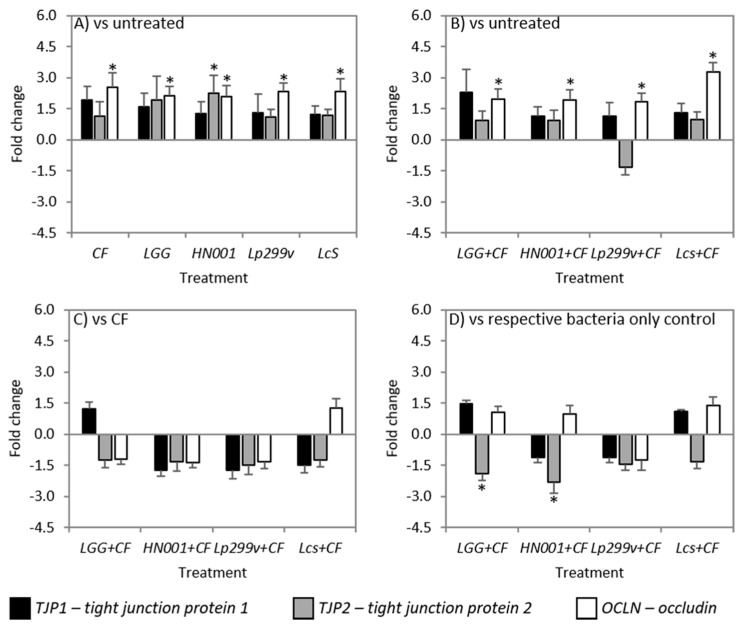
Fold change of *TJP1*, *TJP2*, and *OCLN* mRNA from Caco-2:HT29–MTX (90:10) co-cultures after 3 h incubation with (**A**) probiotic bacteria or a carbohydrate fraction (CF) relative to untreated co-cultures; (**B**) bacteria/CF combinations compared to untreated monolayers; (**C**) bacteria/CF combinations compared to monolayers incubated with CF; and (**D**) bacteria/CF combinations relative to bacteria only controls. Data are expressed as the mean fold change (±SEM) of three replicates across three independent experiments; *n* = 3 A statistically significant difference in fold change at ±1.5 is indicated by * (*P* < 0.05). LGG = *L. rhamnosus* GG; HN001 = *L. rhamnosus* HN001; Lp299v = *L. plantarum* 299v and LcS = *L. casei* Shirota.

The ability of CF and probiotic lactobacilli either alone or in combination to alter the expression level of three TJ related genes of Caco-2:HT29MTX (90:10) co-cultures was quantified using qPCR. The mRNA expression of *OCLN*, but not *TJP1* or *TJP2*, was increased (fold change >1.5) by all bacterial strains, both alone and when in combination with CF, compared to untreated monolayers (Figure 5A,B), but not when compared to CF (Figure 5C) or their respective bacteria controls (Figure 5D). This result is in accordance with Orlando et al. [77] where incubation of Caco-2 cells with *L. rhamnosus* GG over a 6 h period increased *OCLN* expression levels. Increased expression or abundance of occludin is associated with protection of the epithelial barrier, whilst decreased occludin levels are associated with epithelial barrier dysfunction and increased epithelial permeability [11]. Additionally a study by Yan et al. [78] determined that the colonisation of neonatal mice with *L. rhamnosus* GG resulted in increased claudin 3 mRNA expression and TJP1 membrane localisation in the ileum, in addition to increased proliferation and differentiation of epithelial cells indicating that *L. rhamnosus* GG colonisation is beneficial for intestinal growth and development during early life and promotes intestinal functional maturation and tight junction formation [78]. 

In this study, levels of *TJP1* mRNA did not change as a result of any treatment (Figure 5A–D). This result is similar to those findings of Yang et al. [74] where the expression and abundance of *TJP1* were unchanged in IPEC-J2 cells after incubation with *L. reuteri* I5007. Caco-2:HT29–MTX (90:10) co-cultures incubated with *L. rhamnosus* HN001 had increased levels of *TJP2* mRNA compared to untreated (Figure 5A). Treatment of co-cultures with *L. rhamnosus* GG and *L. rhamnosus* HN001 in combination with CF resulted in decreased (*P* < 0.05) levels of *TJP2* mRNA compared to treatment of co-cultures with bacteria alone (Figure 5D).

Changes to other TJ components not measured in this study may also account for the observed increases in TEER. For example, the intracellular plaque protein cingulin, which binds directly to TJP1 as well as to actin filaments of the cytoskeleton [79], contributed to an increase in TEER of Caco-2 monolayers after treatment with *L. plantarum* MB452 [33]. Other important transmembrane TJ proteins such as claudin 1, also interact directly with TJP1, the upregulation of which was observed in jejunal epithelium of young piglets after treatment with *L. reuteri* I5007 [74]. Similarly, treatment of mice with *L. rhamnosus* GG increased the abundance levels of both claudin-1 and claudin-3 protein [80].

There are different mechanisms through which probiotic bacteria can enhance the intestinal barrier and include:

1. The secretion of bacterial proteins such as the p40 and p75 proteins secreted by *L. rhamnosus* GG [81], leading to the activation of protein kinase C (PKC) and the mitogen activated protein (MAP) kinases, extracellular signal-regulated kinases (ERK) 1/2 [36] and enhanced TJ expression;
2. Increased phosphorylation levels and activation of p38, MAPK, and ERK signalling pathways [82,83] resulting in a reorganisation of the TJ complex and an increase in the expression levels of TJ proteins, following treatment with the probiotic mix *VSL#3* [82];
3. Directly modulating the function of epithelial cells by increasing TEER with a corresponding increase in the expression of *TJP1* and *OCLN* after administration of live probiotic strains such as *L. rhamnosus* GG or *L. plantarum* [77,84]; and
4. An increase in occludin and TJP1 in the vicinity of TJ structures of the duodenum following activation of the Toll-like receptor 2 signalling pathway by *L. plantarum* WCFS1 [11].

Although the direct effects of carbohydrate substrates, such as prebiotics, on the intestinal epithelium have been largely unexplored, a study by Wu et al. [13] demonstrated that prebiotics directly activate PKC, resulting in the induction of select TJs such as *OCLN* or *TJP1*, and as such can directly alter TJ expressions to affect epithelial barrier function. Additionally, prebiotics directly alter kinase activities (the kinome) of IECs to regulate host signalling pathways [85]. This suggests that carbohydrate substrates can directly act on the intestinal epithelium and elicit specific cell signalling responses and directly modulate intestinal homeostasis. Whether the combined effects of probiotic lactobacilli and CF act independently or synergistically on the same pathways for the enhancement of the intestinal barrier needs further exploration.

### 3.6. CF and Bacteria When Cultured Alone or in Combination Had Variable Effects on Mucins

Mucins are an important aspect of the protective capacity of the intestinal barrier. Probiotic bacteria either alone or in association with CF had contrasting effects on mucin gene and protein expression. Although the levels of mucin mRNA were modulated by some bacteria/CF combinations, this did not always translate to concomitant changes in the abundance of the respective mucin proteins.

**Figure 6:**
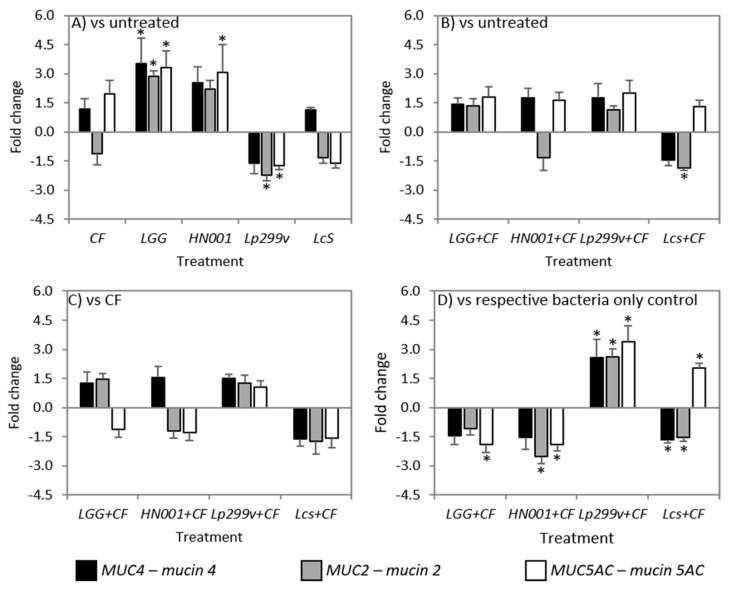
Fold change of *MUC4*, *MUC2*, and *MUC5AC* mRNA from Caco-2:HT29–MTX (90:10) co-cultures after 3 h incubation with (**A**) probiotic bacteria or a carbohydrate fraction (CF) relative to untreated co-cultures; (**B**) bacteria/CF combinations compared to untreated monolayers; (**C**) bacteria/CF combinations compared to monolayers incubated with CF; and (**D**) bacteria/CF combinations relative to bacteria only controls. Data are expressed as the mean fold change (± SEM) of three replicates across three independent experiments; *n* = 3 A statistically significant difference in fold change at ±1.5 is indicated by * (*P* < 0.05). LGG = *L. rhamnosus* GG; HN001 = *L. rhamnosus* HN001; Lp299v = *L. plantarum* 299v and LcS = *L. casei* Shirota.

Treatments were shown to have variable effects on mucin gene expression levels, although CF did not change the levels of any mucin gene investigated compared to untreated co-cultures (Figure 6A). Previously we reported that CF increased *MUC2* and *MUC5AC* gene expression levels in Caco-2:HT29–MTX (90:10) co-cultures [47] after 12 h incubation, whilst a study by Martinez-Augustin et al. [86] reported that levels of *MUC2* and *MUC4* expression was decreased in HT29–MTX cells after exposure to a goat’s milk fraction enriched with oligosaccharides, suggesting increased exposure times of IECs to carbohydrates can have differential effects on mucin gene expression [86]. In contrast, co-cultures incubated with *L. rhamnosus* GG had increased levels of *MUC4*, *MUC2* and *MUC5AC* mRNA compared to untreated co-cultures. An increase in *MUC2* mRNA has previously been shown for the goblet cell line LS174T after 6 h incubation with *L. rhamnosus* GG [87]. This increase resulted from the activation of the EGFR/Akt pathway by the soluble protein p40 produced by *L. rhamnosus* GG, suggesting that this strain does not need to be adhered to the cells to stimulate mucin gene expression. Although Caco-2:HT29–MTX (90:10) co-cultures incubated with *L. rhamnosus* HN001 also had increased levels of all mucin mRNA investigated, only that of *MUC5AC* mRNA was significantly increased compared to untreated (Figure 6A). The expression levels of all mucin genes were similar between co-cultures incubated with *L. casei* Shirota and untreated, whilst incubation with *L. plantarum* 299v resulted in decreased levels of both *MUC2* and *MUC5AC* mRNA (Figure 6A). This result was in contrast to the study of Mack et al. [88] who showed that incubation of HT29 cells with *L. plantarum* 299v increased the expression of both *MUC2* and *MUC3* mRNA. This difference could be attributed to the use of cell co-cultures in this study as opposed to a monoculture of predominantly undifferentiated HT29 cells. Only the combination of *L. casei* Shirota/CF was associated with a decrease in the level of *MUC2* mRNA compared to untreated co-cultures (Figure 6B). This observation may be due to the combined effects of both CF and *L. casei* Shirota, because individually both treatments were noted to cause a decrease in *MUC2* mRNA although these decreases on their own were not significant. All other bacteria/CF combinations had similar levels of *MUC4*, *MUC2* and *MUC5AC* mRNA compared to untreated co-cultures (Figure 6B).

Similar to that observed for TJ related gene expression, there was no difference in the levels of mucin mRNA between any bacteria/CF combinations and CF treated Caco-2:HT29–MTX (90:10) co-cultures (Figure 6C). However, it was of interest that the level of *MUC2* and *MUC5AC* mRNA between CF and the *L. plantarum* 299v/CF treated co-cultures were not different considering the decrease in the expression of these genes after incubation with *L. plantarum* 299v alone. This suggests that the CF when in combination with *L. plantarum* 299v abrogates the detrimental effect of this bacterial strain on the expression of these genes. Although the mechanisms through which CF when in combination with lactobacilli modulates mucin gene expression were not investigated in this study, previous reports have shown that neutral and acidic oligosaccharides from human milk activate EGFR [61], and that activation of EGFR and its downstream targets by probiotic lactobacilli stimulate mucin gene expression [87].

In comparison to co-cultures incubated with *L. rhamnosus* HN001, co-cultures incubated with the *L. rhamnosus* HN001/CF combination had decreased levels of both *MUC2* and *MUC5AC* mRNA. This result indicates that the CF abrogates the beneficial effect of *L. rhamnosus* HN001 in respect to *MUC5AC* mRNA and has an additive detrimental effect on the expression of *MUC2* mRNA (Figure 6D). The combination of *L. casei* Shirota/CF was associated with decreased levels of both *MUC4* and *MUC2* mRNA compared to *L. casei* Shirota alone (Figure 6D), the decrease of *MUC2* which could be attributed to an additive effect of the bacteria and the CF, whilst the decrease of *MUC4* could be viewed as a combined effect. In contrast the levels of *MUC5AC* mRNA were increased for *L. casei* Shirota/CF compared to its bacteria control. Additionally, the levels of all the mucin genes investigated were increased in co-cultures incubated with the *L. plantarum* 299v/CF combination compared to co-cultures incubated with *L. plantarum* 299v alone (Figure 6D), the result of which was not unexpected because *L. plantarum* 299v alone was shown to reduce the expression of all mucin mRNA when compared to untreated co-cultures.

**Figure 7:**
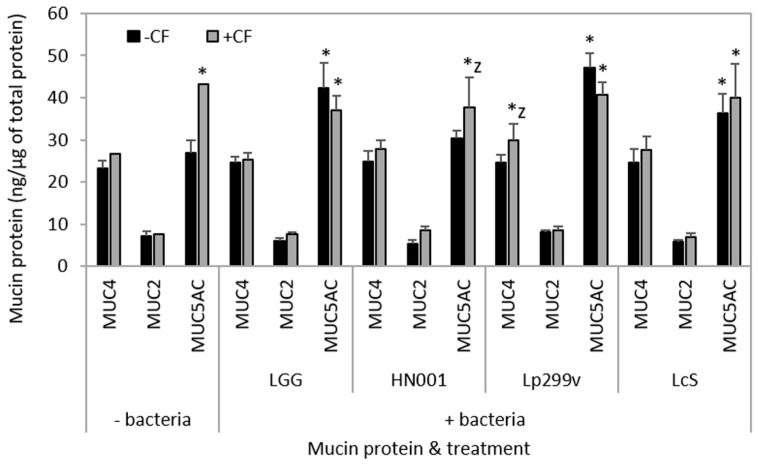
The abundance of MUC4, MUC2 and MUC5AC mucin protein from Caco-2:HT29–MTX (90:10) co-cultures after 3 h incubation with bacteria or a carbohydrate fraction (CF) from caprine milk either alone or in combination. Results are expressed as the mean abundance (±SEM); *n* = 3. * =significantly different (*P* < 0.05) compared to untreated co-cultures, and z = significantly different (*P* < 0.05) compared to co-cultures incubated with the respective bacteria only. UNT = untreated; LGG = *L. rhamnosus* GG; HN001 = *L. rhamnosus* HN001; Lp299v = *L. plantarum* 299v; and LcS = *L. casei* Shirota.

The abundance of MUC2 mucin protein was similar for all monolayers after incubation with any bacteria/CF treatment. In contrast, all bacteria/CF preparations were shown to increase the abundance of MUC5AC compared to untreated co-cultures, except those treated with *L. rhamnosus* HN001. Additionally, monolayers treated with the *L. rhamnosus* HN001/CF combination had an increased relative abundance of MUC5AC protein compared to its respective bacteria control (Figure 7). There was no difference in the abundance of MUC5AC protein between co-cultures incubated with CF and any of the bacteria/CF combinations, but there was a significant increase in the abundance of this protein in CF treated co-cultures compared to untreated (Figure 7). Only Caco-2:HT29–MTX (90:10) co-cultures treated with the combination of *L. plantarum* 299v/CF had an increased MUC4 abundance compared to both untreated and respective bacteria alone co-cultures. However, there was no increase in the relative abundance of MUC4 in co-cultures treated with CF compared to untreated co-cultures (Figure 7). The abundance of MUC4 was similar for all other treatment groups. Wan et al. [89] suggested that the disparity between the changes in the levels of mucin genes and respective mucin proteins levels, such as occurred for the *L. rhamnosus* HN001/CF combination may be attributable to “methods used for quantifying mRNA transcripts levels are more sensitive than those for protein identification and quantification”.

An increase in mucin abundance following incubation with probiotic lactobacilli when compared to untreated co-cultures could potentially enhance their ability to colonise the intestinal tract [39]. However, there was no association between changes in the abundance of specific mucin proteins which resulted in the modulation of bacterial adherence. Such interactions do exist between the lactic acid bacteria, *Lactococcus lactis* subsp. *lactis* BGKP1 and MUC3 and MUC5AC proteins, which aids in the adherence of this bacterial species to the mucus layer [90].

Only the *L. plantarum* 299v/CF combination was associated with an increased abundance of MUC4 mucin protein compared to its respective bacterial control (Figure 7). Membrane-bound mucins such as MUC4, are major components of the glycocalyx, and in addition to their role in providing a physical barrier, are also involved in a wide range of interactions in the luminal environment [91] (such as intracellular signalling events [8]), and play an important role in foetal development, epithelial renewal and differentiation, and epithelial integrity [92,93].

Binding of bacteria to the extracellular domain of membrane mucins can result in cleavage of the mucin. Such cleavage could be an activation signal to the intracellular domain and activation of mucin-specific signalling pathways that alter inflammatory responses, epithelial cell adhesion, and differentiation of epithelial cells [94]. Although the relationship between bacterial binding, cleavage of mucins and activation of the intracellular domain has not been fully elucidated, membrane-bound mucins may act as signalling receptors that sense the external environment and activate intracellular signal transduction pathways essential for barrier maintenance and damage repair [94]. Additionally, the secretion of mucins from goblet cells can be regulated by the host sensing intestinal microbes or their metabolites such as SCFAs or cytokines [95].

## 4. Conclusions

This work demonstrates that probiotic bacteria, when used in combination with CF, are able to increase the barrier integrity to a greater extent than the bacteria or the CF alone, in a Caco-2: HT29–MTX (90:10) co-culture model of the small intestinal epithelium. The precise mechanism through which barrier integrity was increased could not clearly be linked to changes in IEC metabolism associated with CF utilisation, or enhanced mucin gene or protein expression. However, both barrier integrity (TEER) and transcription levels of occludin were enhanced during incubation of co-cultures with bacteria and CF. Global analysis of mRNA and proteins from co-cultures incubated with CF or bacteria alone, and compared to mRNA from co-cultures incubated with CF and bacteria may provide important information on contrasting inter, and intra-cellular signalling cascades and IEC immunomodulation influenced by probiotic bacteria and/or dietary carbohydrates.
